# Bioinspired Collagen/Hyaluronic Acid/Fibrin-Based Hydrogels for Soft Tissue Engineering: Design, Synthesis, and In Vitro Characterization

**DOI:** 10.3390/jfb14100495

**Published:** 2023-10-07

**Authors:** Bianca Bindi, Annalisa Perioli, Priscila Melo, Clara Mattu, Ana Marina Ferreira

**Affiliations:** 1 School of Engineering, Newcastle University, Newcastle upon Tyne NE1 7RU, UK; biancabindi20@gmail.com (B.B.); priscila.melo@newcastle.ac.uk (P.M.); 2 Department of Mechanical and Aerospace Engineering, Politecnico di Torino, Corso Duca degli Abruzzi 24, 10129 Torino, Italy; clara.mattu@polito.it

**Keywords:** collagen, hyaluronic acid, fibrin, hydrogel, spheroid, tissue engineering

## Abstract

A major challenge for future drug development comprises finding alternative models for drug screening. The use of animal models in research is highly controversial, with an ongoing debate on their ethical acceptability. Also, animal models are often poorly predictive of therapeutic outcomes due to the differences between animal and human physiological environments. In this study, we aimed to develop a biomimetic hydrogel that replicates the composition of skin for potential use in in vitro modeling within tissue engineering. The hydrogel was fabricated through the crosslinking of collagen type I, hyaluronic acid, four-arm PEG succinimidyl glutarate (4S-StarPEG), and fibrinogen. Various ratios of these components were systematically optimized to achieve a well-interconnected porosity and desirable rheological properties. To evaluate the hydrogel’s cytocompatibility, fibroblasts were embedded within the matrix. The resulting hydrogel exhibited promising properties as a scaffold, also facilitating the growth of and proliferation of the cells. This biomimetic hydrogel holds great potential for tissue engineering applications, particularly in skin regeneration and cancer research. The study used melanoma spheroids fabricated using the 96-round bottom well plate method as a potential application. The results demonstrate that the developed hydrogels allowed the maintenance of spheroid integrity and viability, meaning it has a promising use as a three-dimensional in vitro model of melanoma for both tissue engineering and drug screening applications.

## 1. Introduction

Regenerative medicine has found a promising avenue in tissue engineering (TE), which aims to restore or replace damaged tissues and organs [1]. Biomimetic hydrogels that closely replicate the composition and properties of native tissues have gained significant attention in this field [1,2,3,4]. The human body’s largest organ, the skin, has a unique hierarchical structure and complex biochemical composition [5]. Hence, there is a crucial need to develop a biomaterial that replicates the native extracellular matrix and biochemical cues of the tissues [6]. Hydrogels can serve as ideal scaffolds for TE applications, including wound healing, skin regeneration, and disease modeling [7].

The extracellular matrix (ECM) of the skin relies heavily on the presence of collagen type I, hyaluronic acid (HA), and fibrin. These biomolecules are crucial for maintaining the structure and functionality of the skin [8]. For instance, collagen type I, as the main structural protein in the skin, provides mechanical stability and guides cellular behavior [9]. Its incorporation into hydrogels promotes cell adhesion, migration, and proliferation. Hyaluronic acid, a natural polysaccharide, contributes to hydration, cell signaling, and tissue lubrication. HA-based hydrogels offer excellent biocompatibility and bioactivity [10]. Fibrin, a natural protein involved in wound healing and clot formation, provides cell adhesion sites and promotes cell infiltration [11]. To create scaffolds for tissue engineering, researchers have been exploring the crosslinking of these biomolecules to develop hydrogels. Four-arm PEG succinimidyl glutarate (4S-StarPEG), a biocompatible crosslinker, facilitates the formation of covalent bonds within hydrogel networks. Its adjustable functional groups enable fine-tuning of the hydrogel properties [12]. Other works have attempted the development of hydrogels utilizing these components individually [13] or in combination with other materials [14,15,16,17,18]. Collagen-based, hyaluronic-based, and fibrin-based hydrogels exhibit poor mechanical properties and fast biodegradability [19,20]. However, the mechanical properties can be increased by physical mixing of these materials or by covalent binding [21]. For example, the introduction of HA within collagen prevents excessive contraction of the hydrogel [22,23], while combining collagen and fibrin can lead to a stiffer hydrogel [17]. However, the optimization of their ratios and the achievement of well-interconnected porosity and rheological properties remain critical challenges. Furthermore, the combination of all these materials into a three-component hydrogel has not been exploited yet, nor has the potential of these hydrogels as scaffolds for specific cell types and their utilization in disease modeling.

Within TE, dermal equivalents can be obtained by encapsulating fibroblasts (FBs) into bovine or rat-tail collagen hydrogels, which have the drawback of including non-human ECM components. Alternatively, other approaches use human decellularized dermis, which replicates the human microenvironment even though donor-dependent variability affects its reproducibility [24,25]. For this reason, collagen-based skin equivalents, embedding FBs, appear to be an optimal solution even though they are subjected to fibroblast-mediated contraction, thus hindering the standardization and reliability of the model for research applications [26]. To overcome this limitation, Lotz et al. developed a full-thickness skin model by embedding FBs into a collagen type I hydrogel chemically crosslinked using four-arm succinimidyl glutarate polyethylene glycol (PEG-SG). The crosslink prevented shrinkage and cellular remodeling by forming stable connections between collagen fibrils [27]. To study diseased skin, dermal equivalents may include melanoma spheroids, which are self-assembled cell aggregates that mimic the tumor microenvironment by recreating the oxygen/nutrient gradient and the necrotic core. This close resemblance to the in vivo situation makes spheroids a great model for drug screening purposes since they mimic the mechanisms that contribute to drug sensitivity and resistance. For example, Muller et al. created melanoma spheroids embedded within a collagen type I hydrogel containing FBs, showing they are a great tool for studying tumor-host interactions [28].

This study aims to address current gaps in ECM development for skin in vitro models by creating a biomimetic hydrogel based on collagen type I, hyaluronic acid (HA), 4S-StarPEG, and fibrin. The hydrogel was optimized for porosity and rheological properties, and its cytotoxicity was assessed by embedding FBs in the hydrogel matrix. To exploit the potential of this hydrogel as a suitable scaffold for culturing skin-based models, this work used melanoma as a testing platform. Melanoma spheroids were created by embedding the cells in the hydrogel and culturing them for 7 days, with cell viability and retainment of shape assessed. The work revealed that this system is a promising platform for in vitro modeling of melanoma, especially in assessing its progression and, in the future, potential therapeutic interventions.

## 2. Materials and Methods

### 2.1. Materials

Type I collagen from rat tails was purchased from R&D Systems (R&D Systems Inc, Minneapolis, MN, USA). Hyaluronic acid sodium salt (53747), 4arm-PEG10K-Succymidyl Glutarate (JKA7031), fibrinogen from bovine plasma (F8630), Phosphate Buffered Saline (PBS), NaOH (1 M), Methylcellulose (M0512), Glycine, and Sodium Dodecyl Sulfate (SDS) were all obtained from Sigma-Aldrich (Sigma-Aldrich, St. Louis, MO, USA). Thrombin bovine high purity grade (154163) was purchased from MP Biomedicals (MP Biomedicals, Irvine, CA, USA). 10X modified essential medium (11430030), RPMI-1640 (11875093), and TNBSA were all purchased from Thermo Fisher Scientific (Thermo Fisher Scientific, Waltham, MA, USA). Dulbecco’s Modified Eagle’s Medium containing [+] 4,5 g/L Glucose, [+] L- Glutamine, [−] L- Pyruvate (41965) was obtained from Gibco life technologies (Thermo Fisher Scientific, Waltham, MA, USA).

### 2.2. Preparation of CHAF Hydrogels

**Figure 1:**
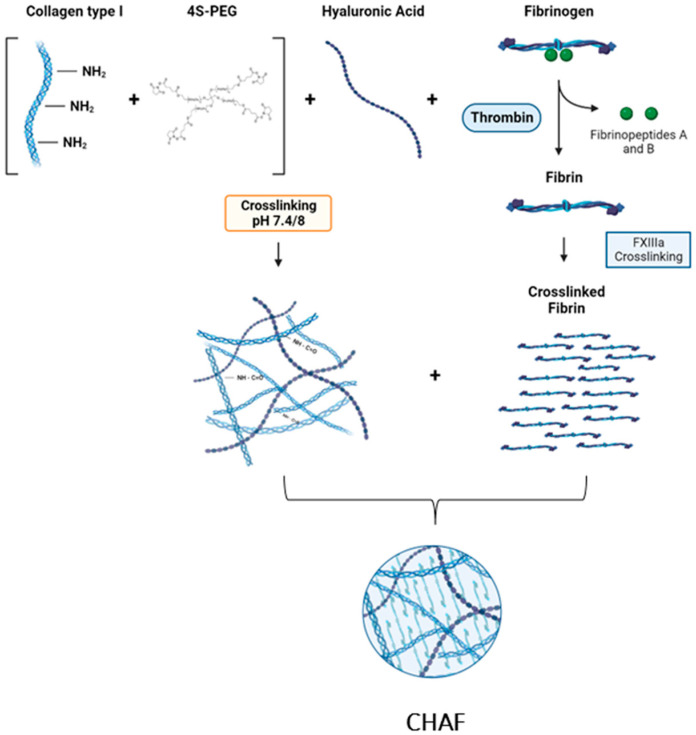
Schematic representation of the collagen type I, hyaluronic Acid, and fibrinogen preparation by incorporating 4S-StarPEG and thrombin as crosslinkers.

The first step consisted of preparing the HA stock solution by dissolving HA powder in 10X modified essential medium (MEM) to a concentration of 100 mg/mL and incubating it overnight at 4 °C. Then, a fibrinogen solution was prepared by dissolving fibrinogen from bovine plasma in PBS to a final concentration of 10% *w*/*v*. To prepare 1 mL of the hydrogel precursor, 100 µL of HA was mixed with 400 µL of collagen (5 mg/mL), using an ice block to avoid physicochemical changes. To neutralize the solution, 18 µL of 1 M NaOH was added, followed by 400 µL of fibrinogen solution and 82 µL of PBS. The collagen was then crosslinked by adding 32 µL of 4S-StarPEG/10X PBS to the previously prepared solution, followed by incubation at 37 °C for 15 min (Figure 1). Finally, 2 U/mL of thrombin was added to polymerize fibrinogen into fibrin. Three different concentrations of 4S-StarPEG solution were studied: 4 mg/mL, 40 mg/mL, and 200 mg/mL to obtain three different formulations. 

CHAF hydrogel formulations are named CHAF_1, CHAF_10, and CHAF_50 (Table 1).

### 2.3. Characterization of CHAF Hydrogels

#### 2.3.1. Quantification of the Crosslinking Degree

The crosslinking degree has been evaluated employing the 2,4,6-Trinitrobenzene Sulfonic Acid (TNBSA) assay, which quantifies the amount of free amine groups in collagen. Firstly, the 0.01% *w*/*v* TNBSA solution was obtained using 0.1 M sodium bicarbonate (pH 8.5) as a diluent, while 10% Sodium Dodecyl Sulfate (SDS) solution was prepared in distilled water (dH_2_O). To promote the reaction, 0.25 mL of TNBSA solution was added to 0.5 mL of each hydrogel sample and incubated at 37 °C for 2 h. Subsequently, 250 µL of 10% SDS solution and 125 µL of 1 M HCl were added to each sample. Hydrogel solubilization was obtained by pipetting the hydrogel and TNBS assay reagents together before absorbance measurement. Next, 200 µL of each sample was transferred into a 96-well plate, and the absorbance was read by a FLUOstar Omega Microplate Reader (BMG Labtech, Ortenberg, Germany) at 335 nm. 

The standard curve was obtained by dissolving Glycine into 0.1 M sodium bicarbonate at different molar concentrations (30 mM, 20 mM, 300 µM, 200 µM, 150 µM, 100 µM, 75 µM, 50 µM, 25 µM, 0 µM). The standards were assessed under the same reaction conditions as the samples.

#### 2.3.2. Morphological Analysis

The morphology of the hydrogel was investigated both after the formation of the gel and at different time points of the degradation test, using an environmental scanning electron microscope (E-SEM) (Scanning Electron Microscope XL30 FEG, Philips, The Netherlands) at an accelerating voltage of 15 kV. The samples were freeze-dried, cut, and fixed on the aluminum stub using carbon conductive tabs and micrographs obtained at different magnifications in different areas of the samples. 

The post-processing of the micrographs via ImageJ software (version 1.52t, Wayne Rasband (NIH), Bethesda, MD, USA) enabled the estimation of the pore size distribution. The pores’ diameter was estimated, assuming that all pores are circular. Frequency distribution of 50 μm width was obtained by grouping pores on three images per sample type. 

#### 2.3.3. Rheological Analysis

Characterization of the rheological properties was performed using a Discovery Hybrid Rheometer HR-2 (TA Instruments, New Castle, DE, USA). The strain sweep test was performed at an angular frequency of 1 Hz to identify the linear viscoelastic region (LVR) and the strain set between 0.01% and 100%. To assess how the loss (G’) and storage (G”) moduli changed with frequency, the amplitude sweep test was performed at increasing angular frequencies ranging from 0.1 to 100 rad/s. The flow ramp test investigated the changes in viscosity (η) at increasing frequencies (0.1 to 100 rad/s). Finally, to assess G’ and G” values and to study the stability of the gel over time, a time sweep test was carried out for 2 h at an angular frequency of 1 Hz and at a constant strain value of 1%. All tests were conducted at 37 °C.

#### 2.3.4. Gelation Time

The gelation time was measured at physiological temperature (37 °C) using the inverting tube test. Briefly, 1 mL of the solution was added into a bijou vial, and the flowability of the sample was analyzed by tilting the vials every 30 s. The time at which the flow of the sample stopped was considered the final gelation time.

#### 2.3.5. Fourier Transform Infrared-Attenuated TR

The chemical composition of the hydrogel was analyzed via infrared spectroscopy using the PerkinElmer UATR Two equipped with diamond total reflectance (ATR) crystal (PerkinElmer Inc., Waltham, MA, USA). The freeze-dried samples were placed under the ATR crystal, and the spectra were analyzed with Perkin Elmer Spectrum software (version 10, PerkinElmer Inc., Waltham, MA, USA) at room temperature, in the range of 4000–500 cm^−1^. All the obtained spectra resulted from an average of 16 scans at a resolution of 4 cm^−1^.

#### 2.3.6. Nutrient Diffusion

The diffusion of the nutrients through the hydrogel was tested using a qualitative and quantitative analysis to evaluate its permeability. A glucose solution was prepared by dissolving in PBS the light-sensitive 2-NBDG (2-(N-(7- Bitrobenz-2-oxa-1,3-diazol-4-yl) Amino)-2-Deoxyglucose) at a concentration of 0.06845 mg/mL according to the protocol proposed by Ribeiro et al. [29]. The qualitative analysis was performed by jellifying 2 mL of the solution in a bijou vial, followed by the addition of 500 µL of 2-NBDG solution and observing its diffusion over time. The time to obtain a complete yellow color gel corresponds to the nutrient’s absorption time. A quantitative analysis was performed on lyophilized samples. Briefly, samples were weighed, washed with PBS, and positioned in a 48-well plate. Then, 1 mL of the glucose solution was added to each well, and the plate was incubated in a light-protected environment. At each time point (15 min, 30 min, 1, 4, and 24 h), 200 µL of each well solution was transferred to a 96-well plate and read at the FLUOstar Omega Microplate Reader (BMG Labtech) (465 nm excitation/540 nm emission). During the experiment time, the glucose solution and the well plates were protected from light.

#### 2.3.7. Water Uptake and Water Content

Samples were frozen at −20 °C overnight and then lyophilized for 48 h in a freeze-dryer (Alpha 1-2 LDplus, CHRIST, Osterode am Harz, Germany) to remove all the water content. Lyophilized hydrogel samples were weighted and placed separately in a bijou vial containing 5 mL of PBS and stored at 37 °C inside the incubator (Thermo Fisher, Waltham, MA, USA). The weight of all the samples was measured before immersion (initial weight of the hydrogel, dry) and after 5, 15, 30, 45 min, 1, 2, 4, and 24 h of incubation in PBS. At each time point, the samples were weighed after gently drying the extra PBS on the surface using tissue paper. The water uptake (WU) percentage was evaluated using the following equation:

WU (%) = 100 (W_t_ − W_i_)/W_i_ (1)

where W_i_ is the initial weight of the hydrogel (dry), and W_t_ is the weight of the hydrogel at the specific time point.

The percentage of water content was calculated using the following equation:

Water content (%) = 100 (W_t_ − W_i_)/W_t_ (2)

where W_i_ is the initial weight of the hydrogel (dry), W_t_ is the weight of the hydrogel at the specific time point.

#### 2.3.8. Degradation

Freeze-dried samples were weighed and then immersed in 5 mL PBS (pH 7.4) and incubated at 37 °C for up to 7 days. At each time point (1, 3, 24, 48 h, 5, and 7 days), the samples were taken out from the bijou vial, rinsed with water, frozen, and lyophilized. The percentage of degradation was calculated using the equation:

Weight Loss (%) = 100 (W_i_ − W_Dt_)/W_i_ (3)

where W_i_ is the initial weight of the hydrogel (dry), and W_Dt_ is the weight of the hydrogel (dry) at the specific time point.

### 2.4. Preparation of Sterile CHAF Hydrogels

The CHAF_1 samples were prepared in sterile conditions by adding the hydrogel precursor into a 96-well and replacing PBS with DMEM in its formulation. HA powder, MEM, collagen solution, fibrinogen powder, 4S-StarPEG, and thrombin were provided sterile from the supplier. CHAF_1 preparation followed the protocol reported in Section 2.2.

### 2.5. Cytocompatibility and Biological Characterisation Using Human Fibroblasts

Normal Human dermal fibroblasts NHDF-c adult cells (FBs) were purchased from PromoCell (Sigma Aldrich, St. Louis, MO, USA) at passage 2 and cultured according to general protocols. Cells were used for cell culture at passage 5. Briefly, cells were maintained in high glucose DMEM supplemented with 10% fetal bovine serum (FBS) (Sigma Life Science, St. Louis, MO, USA) and 1% penicillin/streptomycin (P/S) (Sigma Life Science) and kept in a humified Midi 40 incubator (Thermo Fisher, Waltham, MA, USA) at 37 °C with 5% CO_2_. Upon 80% confluency, cells were detached using trypsin/EDTA (0.25% *w*/*v* trypsin/0.02% EDTA, Gibco) for cell seeding.

#### 2.5.1. 2D Seeding and Embedding of Fibroblasts into CHAF_1 Hydrogel

For the seeding of cells in two dimensions (2D) (on top of the hydrogel), the hydrogel was prepared and allowed to settle by incubating it without media at 37 °C for 15 min. Subsequently, 10,000 cells were added to each well and covered with DMEM. For the embedding within the hydrogel, cells were added to the gel precursor at a density of 500,000 cells/mL and gently mixed. The crosslinkers (4S-StarPEG and thrombin) were then added to allow the formation of the hydrogel. For both experiments, cells seeded on tissue culture plastic were used as a control (10,000 cells/well). The samples were cultured for 7 days, and the media was refreshed every 48 h. 

#### 2.5.2. PrestoBlue Assay

The metabolic activity of FBs in both experiments was evaluated by performing PrestoBlue assay on days 1, 3, and 7. Briefly, the PrestoBlue™ Cell viability reagent (Invitrogen, Thermo Fisher Scientific, Waltham, MA, USA) was warmed up at room temperature and diluted in DMEM at a 1:10 ratio. The culture media was removed, and each sample was washed with pre-warmed PBS, followed by the addition of 0.5 mL of PrestoBlue solution and incubation at 37 °C with 5% CO_2_ for 2 h, protected from light. The supernatant was transferred into a 96-well plate, and the fluorescence was measured by the FLUOstar Omega Microplate Reader (BMG Labtech) at excitation of 560 nm and emission of 590 nm. Samples were then washed twice with PBS to remove residues of PrestoBlue solution, and fresh media was added. The experimental values were corrected by subtracting the average fluorescence of the PrestoBlue stock, which was used as a control. The test was performed in triplicate, and the results were treated with GraphPad Prism™ 9 software (GraphPad Software, La Jolla, CA, USA).

#### 2.5.3. Live and Dead Assay

Cell viability was assessed using the Live/Dead Cell Double Staining (Sigma Aldrich) on days 1 and 3. Briefly, the staining solution was prepared by adding 10 µL of Calcein-AM solution to 5 µL propidium iodide solution in 5 mL PBS, as per manufacturer instructions. The samples were washed with PBS and then incubated in the staining solution for 30 min at 37 °C, 5% CO_2,_ protected from light. The solution was removed, and the samples were imaged using an EVOS M5000 microscope (Thermo Fisher Scientific). 

#### 2.5.4. Cell Morphology by Fluorescence Microscopy

The cell morphology of fibroblasts in contact or within the gel was performed on days 1 and 7. At each time point, samples were washed with PBS and fixed in 4% *w*/*v* paraformaldehyde (PFA) (Thermo Scientific) for 30 min at 37 °C with 5% CO_2_. Samples were then permeabilized with a mix of 0.1% *v*/*v* Tween20^®^/PBS and incubated in a solution of ActinRed 555 ReadyProbes/PBS (Invitrogen, Thermo Fisher Scientific) for 30 min at 37 °C with 5% CO_2_. Finally, the samples were washed in PBS, and 1 drop of Fluoroshield™ with DAPI (Sigma Life Science) was added to the top of each sample. Finally, the samples were flipped into a glass cover slip and imaged using an EVOS M5000 microscope (Thermo Fisher Scientific). 

### 2.6. Formation of Melanoma Spheroids and Culture within the CHAF_1 Hydrogel

The human melanoma cell line 451-Lu was purchased from Rockland Scientific (Philadelphia (PA), USA) at passage 38 and maintained in RPMI-1640 supplemented with 10% FBS and 1% P/S and kept in a humified incubator at 37 °C with 5% CO_2_. After the expansion, cells were used between passages 39 and 43 for spheroids fabrication via Round-bottom 96-well plates. Firstly, methylcellulose powder was dissolved in RPMI-140 media at a final concentration of 0.25% *w*/*v* and then passed through a 0.22 μm filter to be sterilized. The 451-Lu cells were then washed in PBS and detached using Trypsin-EDTA in PBS and centrifuged at 12,000 rpm. The cell pellet was re-suspended in RPMI media supplemented with 0.25% methylcellulose (prepared as previously described) at a density of 5 × 10^3^, 10 × 10^3^, 25 × 10^3,^ and 50 × 10^3^ cells per 150 μL of medium. Round-bottom 96-well plates (non-treated) were seeded with different cellular concentrations, and the plates were incubated at 37 °C, 5% CO_2_ for 72 h to allow aggregation. Upon successful aggregation, the spheroids were maintained in media.

After 4 days of culture, the 451-Lu spheroids were integrated into CHAF_1 hydrogel. Media was carefully removed from the well, and spheroids were aspirated in the smallest volume possible of media using a 1 mL pipette tip, with the ends cut off to make them wider. The spheroid was then placed over the hydrogel precursor solution, and the crosslinkers were subsequently added and slowly mixed to allow the formation of the gel and avoid spheroid disruption. To allow the spheroids to settle, the hydrogel was incubated without media for 30 min at 37 °C, 5% CO_2_. Afterward, each gel was covered with 200 μL of fresh RPMI/10% FBS/1%P/S media and culture for 7 days, with media refreshed every two days. Spheroids maintained in media were used as control.

#### 2.6.1. CellTiter-Glo^®^ 3D Cell Viability Assay

To determine the number of viable cells in the spheroids embedded in CHAF_1 hydrogel, the CellTiter-Glo^®^ 3D Cell Viability Assay (Promega, Madison, WI, USA) was performed according to the manufacturer’s instructions. Firstly, the CellTiter-Glo^®^ 3D reagent was thawed overnight at 4 °C. Then, the reagent was equilibrated to room temperature and added to the samples in a volume ratio of 1:1. The content of the wells was mixed vigorously to induce cell lysis and obtain a homogeneous solution. The mixture was then transferred to an opaque-walled 96 well-plate and incubated for 25 min. The recording was performed in luminescence with a filter-based multimode microplate reader (FLUOstar Omega, BMG Labtech, Ortenberg, Germany). The experimental values were corrected by subtracting the average luminescence of the CellTiter-Glo^®^ 3D reagent used as a control. Data were analyzed using GraphPad Prism^®^.

#### 2.6.2. Morphological Analysis of 3D Cultures

Morphology and growth of the spheroids were monitored on days 3, 5, and 7, targeting changes in shape and area. Micrographs were taken using an EVOS M5000 microscope (Thermo Fisher Scientific) in Brightfield, and ImageJ software was used to obtain morphological 2D parameters (area and diameter).

### 2.7. Statistical Analysis

Tests were performed on triplicates of each sample, and results were presented as mean value ± standard deviation or standard error. One-way ANOVA with Bonferroni post hoc test was performed with GraphPad Prism™ 9 software (GraphPad Software, La Jolla, CA, USA) using a level of significance of *p* < 0.1 (*), *p* < 0.05 (**), *p* < 0.001 (***), and *p* < 0.0001 (****).

## 3. Results

### 3.1. Characterization of Collagen/Hyaluronic Acid/Fibrin (CHAF) Hydrogels

Three different CHAF hydrogel formulations have been obtained by using different collagen to 4S-StarPEG ratios, as presented in Table 1. Then, a TNBSA assay was performed to evaluate the crosslinking efficacy and to optimize the C/PEG ratio. 

**Figure 2:**
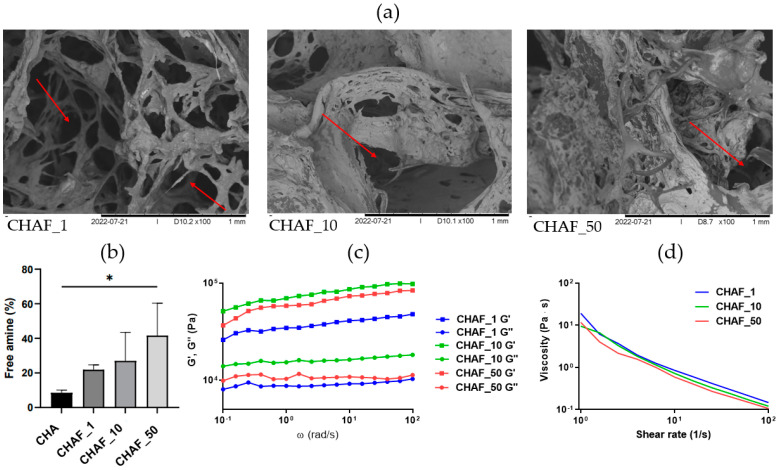
(**a**) Scanning electron microscopy images representing cross-section microstructure of CHAF hydrogel with different collagen to 4SU−StarPEG ratios. Red arrows highlight pores with the same size in the different samples. (**b**) Quantitation of the crosslinking degree via TNBSA assay (data points indicate mean ± SD, *n* = 3), *p* < 0.1 (*). (**c**) Frequency sweep of CHAF hydrogels with different collagen to 4S−StarPEG ratio (1:1, 1:10, and 1:50); (**d**) Flow ramp of CHAF precursor solution with different collagen to 4S−StarPEG ratio (1:1, 1:10, and 1:50).

E-SEM analysis was performed on the three different hydrogel formulations to evaluate the influence of the collagen-to-PEG ratio on the CHAF hydrogel morphology (Figure 2a). While CHAF_1 showed a highly porous structure and interconnected porosity, CHAF_10 and CHAF_50 presented very little number of pores, with no apparent interconnection, therefore not able to ensure the right transport of oxygen, nutrients, and waste, nor the migration of cells, all necessary for their survival. 

Overall, as presented in Figure 2b, all hydrogel formulations exhibit a low percentage of free amine groups, meaning that the crosslinking efficacy is high. In detail, the percentage of free amine groups was 21.9% ± 2.8, 27.0% ± 16.4, and 41.6% ± 18.7 for CHAF_1, CHAF_10, and CHAF_50, respectively. This shows that all the different formulations have an efficacy of crosslinking over 60%.

Rheological analysis was performed to evaluate the viscoelastic properties of CHAF hydrogels. The frequency sweep (Figure 2c) was carried out on CHAF_1, CHAF_10, and CHAF_50 to observe how the loss (G″) and storage (G′) moduli changed with frequency (Table 2). CHAF_1 exhibited the lowest G′ and G″ values, while CHAF_10 had the highest ones. From the strain sweep test, it is noticeable that, for each formulation, G’ and G” moduli start decreasing when strain increases, approximately after 10% strain. As expected, for higher strains, the G′ decreases significantly. These observations are applicable to each formulation. The flow ramp test (Figure 2d) reported a shear-thinning behavior for all tested solutions. No differences were observed among the three formulations. 

The CHAF_1 hydrogel was considered the most promising candidate for the desired application, bioprinting, and hence was taken forward for further studies. 

**Figure 3:**
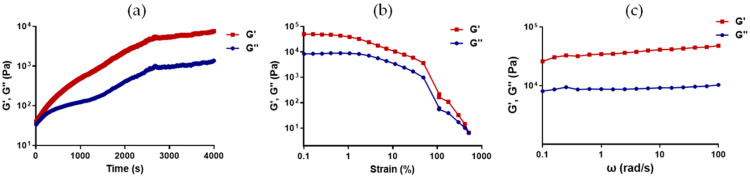
Rheological analysis of CHAF_1 sample. (**a**) Time sweep. (**b**) Strain sweep. (**c**) Amplitude sweep.

The stability in physiological conditions was evaluated via a time sweep test (Figure 3a), which showed that the hydrogel became stable after 45 min from the addition of the crosslinkers. The strain sweep (Figure 3b) allowed the identification of the linear viscoelastic region (LVR), while the frequency sweep (Figure 3c) showed the G″ and G′ moduli under different physiological frequencies. The G′ and G″ values were respectively found to be 37.6 kPa and 9.1 kPa.

**Figure 4:**
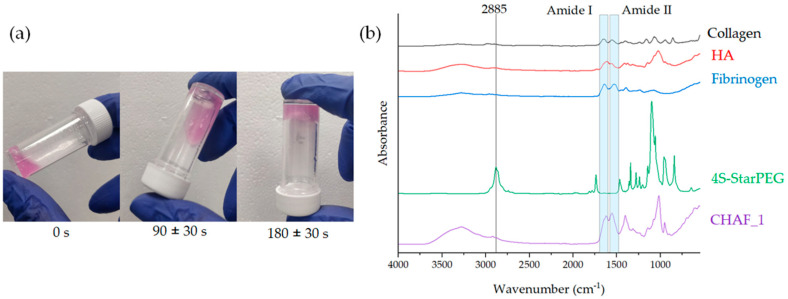
(**a**) Tube inverting method of CHAF_1. (**b**) ATR−FTIR spectra of collagen, hyaluronic acid (HA), fibrin, 4S−StarPEG (PEG), and CHAF_1 hydrogel.

The gelation times were assessed via an inverting tube test, which demonstrated that CHAF_1 became a gel within 3 min ± 30 s (Figure 4a). To evaluate the functional group and chemical bonding present in the developed hydrogel, ATR-FTIR was performed (Figure 4b) for each of the individual components of the gels (collagen, HA, fibrin, and 4S-StarPEG) as well as freeze-dried CHAF_1. A complete list of peaks is presented in Table 3.

In particular, the CHAF_1 spectrum exhibits a peak at 1640 cm^−1^ due to the amide bond (NH–C=O) formation. This amide bond formation occurs due to the interaction between amine groups of collagens with succinimidyl ester groups of 4S-StarPEG [41]. 

**Figure 5:**
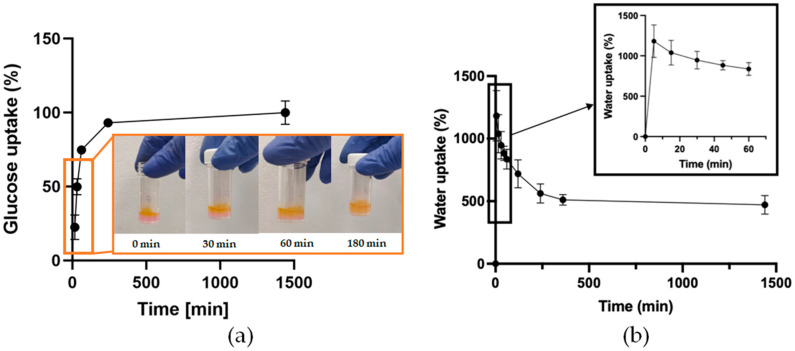
(**a**) Glucose uptake analysis of CHAF_1 at different time points [15, 30 min, 1, 4, and 24 h] with the qualitative analysis of the glucose solution diffusion throughout the CHAF_1 hydrogel up to 3 h. Time points are connected with lines to show change over time. (**b**) Measurement of the water uptake ability of CHAF_1. The graph shows the percentage of water uptake of the samples for 24 h. Data points indicate mean ± SD, *n* = 3. Time points are connected with lines to show change over time.

Subsequently, the 2-NBDG glucose uptake test was used to investigate nutrient uptake and diffusion within the CHAF_1 hydrogel (Figure 5a). This quantitative method demonstrated that in 1 h, nearly 50% of the glucose solution was absorbed by the hydrogel. Moreover, a qualitative analysis showed that nutrient diffusion through the hydrogel via glucose dye solution absorption occurred in 3 h when the gel presented a homogeneous yellow color.

The water uptake test was then conducted in order to assess the percentage of absorbed water by the freeze-dried samples immersed in PBS (pH 7.4) at 37 °C for up to 24 h (Figure 5b). CHAF_1 exhibited a rapid initial water uptake within 15 min up to the 1182.1% ± 201.9. Then, the samples started releasing some of the water, reaching a value of approximately 562.0% ± 76.0 within 4 h. After the stabilization, a water uptake plateau can be observed within 24 h of analysis. At the plateau, the water content has been calculated, revealing a water content of 71.8 ± 12.7%, which is comparable with the water content of the native skin tissue (Table 4).

**Figure 6:**
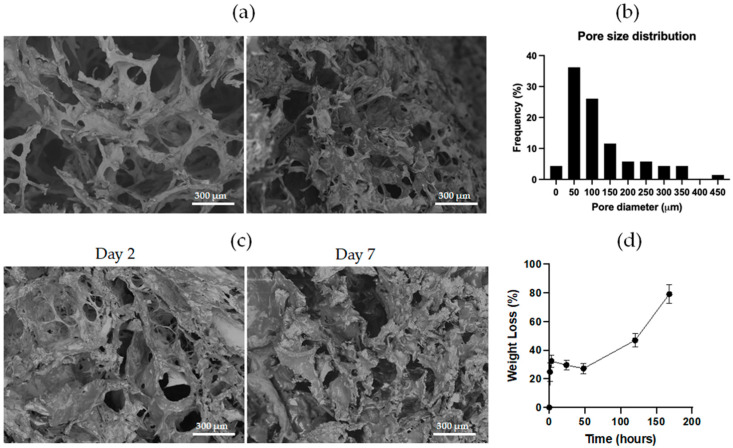
(**a**) Scanning electron microscopy images representing the cross-section microstructure of CHAF_1 hydrogel. (**b**) Pore size distribution of CHAF_1. (**c**) Micrographs representing the cross-section microstructure of CHAF_1 hydrogel at different time points of the degradation test. (**d**) Measurement of the in vitro degradation of CHAF_1. The graph shows the degradation measured through the weight loss during 7 days of immersion in PBS. Data points indicate mean ± SD, *n* = 3. Time points are connected with lines to show change over time.

To evaluate the internal morphology of the CHAF_1 sample, E-SEM analysis was performed (Figure 6a). From the post-processing of the micrographs, pore size and distribution were calculated (Figure 6b). CHAF_1 has almost 40% of its pores in the range of 50 μm with a hierarchical porosity ranging from 17 μm to 435 μm. The changes in the morphology of CHAF_1 were also analyzed during the degradation test (Figure 6c). Differences were found in the hydrogel appearance in the two analyses. In detail, the average pore size rose during degradation from 120 ± 97 μm at day 0 to 169 ± 82 μm at day 7 (Table 5).

The measurement of the in vitro degradation showed that the weight lost by the CHAF_1 sample increased rapidly, reaching roughly 30% in 3 h (Figure 6d) and reaching 80% by day 7, showing that the material is mostly degraded. 

### 3.2. Collagen/Hyaluronic Acid/Fibrin (CHAF_1) Cytocompatibility Assessment

To evaluate the hydrogel’s ability to support cell viability, FBs were seeded on top of CHAF_1, and Live and Dead assay was performed. 

**Figure 7:**
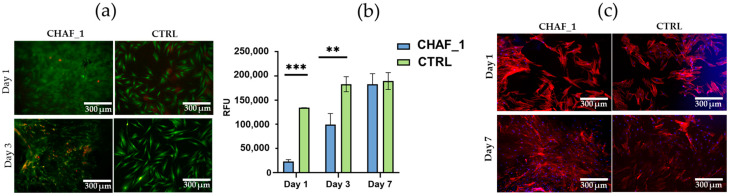
(**a**) Live/dead stained images of fibroblasts seeded on top of CHAF_1 and cells on tissue culture plastic as control (CTRL). Imaging was performed on days 1 and 3. Live cells are stained in green, and the dead cells in red (10× magnification). (**b**) PrestoBlue assay results for fibroblasts seeded on the top of CHAF_1 and on tissue culture plastic as control (CTRL) at 1, 3, and 7 days. RFU refers to relative fluorescence units. Data represent average ± SD (*n* = 3), *p* < 0.01 (**), and *p* < 0.001 (***). (**c**) DAPI/Phalloidin stained images of fibroblasts seeded on top of CHAF_1 and cells on tissue culture plastic as a control (CTRL). Imaging was performed on days 1 and 7. DAPI stains nuclei in blue, and phalloidin stains cytoskeletons in red (10× magnification).

As shown in Figure 7a, a few dead cells (red stained) can be observed in all samples on days 1 and 3, but overall, results highlight that the cells remained viable over time (green). Moreover, as shown from the Presto Blue assay (Figure 7b), cells seeded over CHAF_1 showed a significant increase in their metabolic activity and maintained a spindle-like morphology (typical of FBs), similar to the CTRL (Figure 7c).

**Figure 8:**
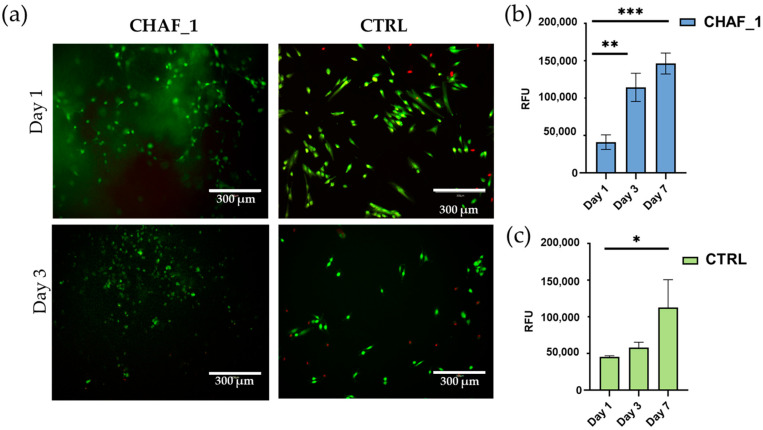
(**a**) Live/dead stained images of FBs encapsulated within CHAF_1 and cells on tissue culture plastic as a control (CTRL). Imaging was performed on days 1 and 3. Live cells are stained in green, and the dead cells in red. (**b**) PrestoBlue assay results for FBs encapsulated within CHAF_1 at 1, 3, and 7 days. (**c**) FBs seeded on tissue culture plastic are used as a control (CTRL). Data represent average ± SD (*n* = 3), *p* < 0.05 (*), *p* < 0.01 (**), and *p* < 0.001 (***).

The fibroblasts’ cellular viability was also evaluated when encapsulated within the hydrogel. Results show that cells were alive after 3 days of culture (Figure 8a) and proliferated up to 7 days (Figure 8b), similar to the results obtained for the CTRL (Figure 8a,c). Given the PrestoBlue assay mechanism of action, when analyzing three-dimensional (3D) structures, the solution is only able to diffuse in the most superficial layers. Choosing 500,000 cells/mL for the embedded samples and 10,000 cells for the CTRL allowed us to compare the results of the test.

### 3.3. Melanoma Spheroids Morphological Analysis and Encapsulation

To evaluate the optimal cell density to form spheroids, a morphological evaluation was conducted. 

**Figure 9:**
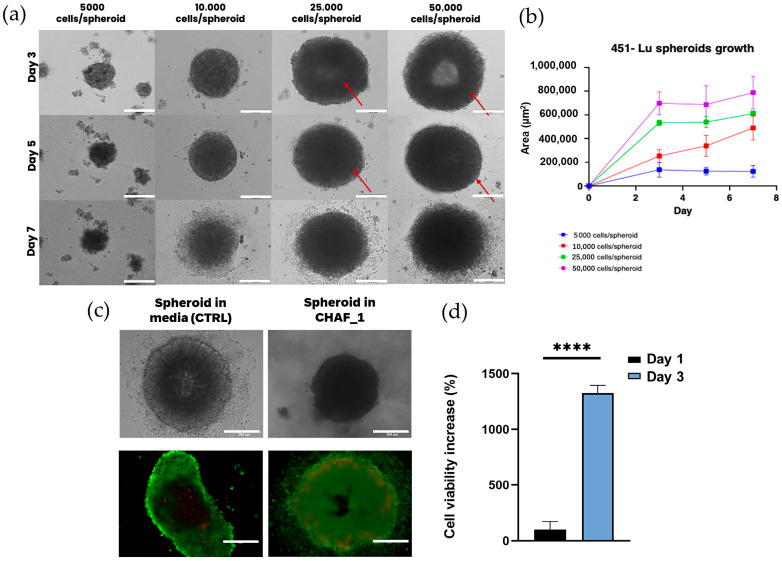
(**a**) The Brightfield microscopy images show the evolution in 451-Lu spheroid morphology at different time points (3, 5, and 7 days) when seeded at different cell densities (5000, 10,000, 25,000, 50,000 cells/spheroid). Scale bar = 300 μm. Red arrows highlight the compactness of the spheroids over time. (**b**) Change in the 451-Lu spheroid area over 7 days for four different initial seeding densities (5000, 10,000, 25,000, and 50,000 cells/spheroid). Data points indicate mean ± SD, *n* = 3. (**c**) Brightfield microscopy and live/dead stained images (fluorescence microscopy) of the 451-Lu spheroid (25,000 cells/spheroid) embedded within the CHAF_1 hydrogel and in media as a control (CTRL). Images were taken after 24 h from the encapsulation. Scale bar = 300 µm. (**d**) Cell viability measured with CellTiter Glo assay. Data points indicate mean pm SEM, *n* = 3, *p* < 0.0001 (****).

Spheroids culture in the TCP were captured using a light microscope image (Figure 9a), and these images were used to calculate their size and area. Notably, spheroid size is cell-dependent; as the cell density increases, the area of the spheroid grows. However, the growth pattern observed does not seem to be proportional to the initial cell density; in fact, an increasingly higher number of cells produced denser spheroids. Moreover, cell density seems to affect the ease of spheroids formation, which occurs more frequently when using 25,000 and 50,000 cells. The measurements performed with ImageJ showed that spheroid diameters range from 400 to 1000 μm (Figure 9b), corroborating the abovementioned data.

On day 4, after the initial spot, 451-Lu spheroids were encapsulated by pipetting these spheroids in the CHAF_1 hydrogel. Images of the spheroid within the hydrogel and in media were taken by Brightfield microscopy. From Figure 9c, it is evident that CHAF_1 provides a matrix that can hold the spheroids well, preventing their disruption, whereas, in media, the cellular aggregates tend to open. Live/dead staining (Figure 9c) was performed to visualize the distribution of dead cells within the hydrogel. The presence of dead cells (red stained) in the central part of the spheroids indicates the formation of an apoptotic core, which can also be observed for the CTRL. On the other hand, the outer layers of the spheroids are characterized by proliferating viable cells (in green). A closer observation of these images suggests that, within CHAF_1, the peripheral cells appear to adopt an invasive behavior. As also seen in Figure 9a, the spheroidal shape of the cellular aggregates is not preserved when the spheroid is immersed in media, which highlights CHAF_1’s suitability as a hosting matrix for melanoma spheroids.

Finally, cell viability within the spheroids was assessed through the CellTiter-Glo^®^ 3D assay. In the graph shown in Figure 9d, a significant increase in the percentage of viable cells at day 3 for spheroids in CHAF_1 related to their viability at day 1 (100%) can be observed. 

## 4. Discussion

The hydrogel presented in this work, namely CHAF_1, has been designed following three main requirements: biomimetic composition, rheological properties resembling human skin, and interconnected and hierarchical porosity. Primary human fibroblasts were embedded within the CHAF_1 to assess the cytocompatibility of the material. To explore the potential of CHAF_1 as an ECM-like material for skin TE, melanoma spheroids were created within the hydrogel, as this strategy is currently considered the most suitable model to mimic metastatic melanoma. Such a model can further be used for studying the interactions occurring between melanoma cells and other cell types as well as the main components of human skin. 

Given that the main components of the skin are collagen type I and HA, the hydrogel formulations presented in this work contain collagen type I, HA, and fibrin. The 4S-StarPEG is used as a crosslinker where its NHS groups crosslink collagen amino groups under physiological conditions [12], while hyaluronic acid is likely entrapped within the hydrogel primarily through physical and ionic interactions, and its release kinetics may be influenced by the steric hindrance of high molecular weight hyaluronic acid. To assess the degree of crosslinking, TNBSA was performed on three different formulations, with changing collagen:4S-StarPEG ratios, revealing that the highest degree of crosslinking was obtained at a ratio of 1:1. Increasing the PEG ratio (CHAF_10 and CHAF_50) did not improve the crosslinking efficiency, probably due to a steric hindrance caused by a saturation of 4S-StarPEG [12]. To investigate the potential of this material for extrusion bioprinting, rheological testing was performed to assess the extrudability and stability of the solutions via flow ramp and time sweep tests, respectively. The time sweep test showed that the hydrogel remains stable for 2 h, which is an acceptable printing time for hydrogel-based materials [43]. The flow ramp revealed a decrease in viscosity with increasing shear rate, meaning the material is shear thinning, hence extrudable [9]. Furthermore, the shear-thinning behavior is crucial to preserve cell viability during the printing process, as elevated shear rates may damage cells [44]. The morphology of the obtained hydrogels from different CHAF formulations revealed that CHAF_1, unlike the other formulations, exhibited well-interconnected porous structure and hierarchical porosity, necessary for cell survival since it ensures oxygen, nutrients, and waste diffusion, as well as cell migration [45]. These results, alongside the rheological evaluation for potential bioprinting, led to the choice of CHAF_1 as the most suitable hydrogel formulation for this application; therefore, a deeper characterization was performed on it, including the biological assessment. 

The in-depth analysis of CHAF_1 started by investigating its viscoelastic properties at increasing strain. The obtained values for G′ and G″ moduli were approximately 40 and 10 kPa, respectively, thus resembling human skin, which ranges from 1–10 kPa [46]. CHAF_1 gelation time was assessed using the tube inverting test. Its gelation occurred in 3 min ± 30 s, which is comparable with other collagen/hyaluronic acid hydrogels [47] and fibrin hydrogels using the same amount of thrombin [48]. This fast gelation time is desired for in situ forming hydrogel to rapidly encapsulate cells and avoid their sedimentation [45,47]. For bioprinting applications, namely the use of the hydrogel as a bio-ink, the presence of two different crosslinkers (4S-StarPEG and thrombin) allows the modulation of the hydrogel gelation time. Since 4S-StarPEG and thrombin have different crosslinking kinetics, we hypothesized to print the formulation containing collagen, hyaluronic acid, fibrinogen, and 4S-StarPEG in a thrombin-containing bath [49,50]. The thrombin’s fast crosslinking enables the instant jellification of the extruded filaments, which can retain their shape and geometry, allowing the deposition of other filaments above, forming a 3D structure with high shape fidelity [50]. The sacrificial bath can then be removed, depending on the material used (e.g., gelatin by placing at 37 °C, alginate by enzymatic digestion). FTIR-ATR analysis further verified the occurred crosslinking. The obtained spectra presented all the component’s typical peaks (Table 3), in particular, the peak at 1640 cm^−1^ due to the amide bond formation, a result of the interaction between NHS groups on 4S-StarPEG and amine groups in collagen [12,41]. From the water uptake test, CHAF_1 absorbed more than 1000% of PBS in the first 15 min and released about half of it in the following 4 h. The high amount of water absorbed is typical of natural biomaterials [51], and the high amount of HA in the formulation boosted the water absorption [52]. The water uptake is also related to the characteristics of the external solution. For instance, the water uptake of hydrogels in salt solutions decreases compared to distilled water [53]. However, at the equilibrium stage, the water content of the hydrogel was calculated. CHAF_1 showed approximately 71.8% ± 12.7% of water content, which is close to human dermis water content [42]. These outcomes suggest that the hydrogel may closely resemble human skin and provide a suitable environment for host cells [54]. In addition, to further evaluate the hydrogel’s ability to allow the transport of nutrients and waste, a 2-NBDG uptake test was performed. The qualitative and quantitative analysis revealed that the nutrients were completely absorbed within 3 h, with about 50% of nutrients absorbed within the first 30 min. The ability of the hydrogel to rapidly absorb nutrients is crucial to sustain cell viability when cells are embedded within the hydrogel structure. This result suggests that the morphological structure of the CHAF_1 ensures the transport of oxygen and nutrients, allowing for cell survival [44]. This is further supported by the results of E-SEM image analysis, which demonstrated CHAF_1 suitability for supporting cell growth and proliferation due to a hierarchical porosity with pores from 17 μm to 435 μm, with an average pore size of 120 µm. CHAF_1 degradation profile remains under 30% during the first 48 h and increases to 80% after 7 days. As observed in SEM images, the pore size increases significantly as the hydrogel degrades. The fast degradation rate is related to the natural origin of the hydrogel components [55,56]. However, the relatively rapid degradation rate of the CHAF hydrogel within 7 days can be compensated by the high proliferative nature of FBs that secrete high amounts of type I collagen and HA [57]. Nevertheless, these hypotheses need further work to be confirmed. This degradation rate is similar to other analogous studies and is acceptable for the application of hydrogel as a skin equivalent for drug delivery or wound healing applications in vivo [58,59,60]. Nonetheless, in the future, the degradation rate must be assessed in the presence of enzymes (such as hyaluronidase or collagenase), and its properties must be tuned to ensure longer stability over time.

The cytocompatibility of the CHAF_1 hydrogel was investigated by seeding FBs on the surface of the hydrogel. Live/dead staining images show that cells were viable when in contact with the hydrogel, with a significant increase in their metabolic activity over the time of incubation. This evidenced that the hydrogel and crosslinker used in this work, namely 4S-StarPEG, did not produce any cytotoxic effects, as reported by other studies in vitro and in vivo [61,62,63]. Further, the metabolic activity of fibroblasts seeded on CHAF_1 hydrogel was significantly higher over time when compared to cells onto tissue culture plastic (CTRL), demonstrating the importance of a biomimetic environment provided by the naturally derived biopolymers. Indeed, suitable cell microenvironments and biodegradation support cell function, including their growth, proliferation, and remodeling of the surrounding matrix (e.g., producing ECM and the formation of neo-tissue) [57]. The immunostaining analyses (Dapi—nucleus, Phalloidin—actin filaments/cytoskeleton) show cells widely spread out and adhered to the CHAF_1 surface, displaying a typical spindle-like morphology. FBs are a key cellular population in the dermis, and the hydrogel impact of their fate is also assessed when these are encapsulated. Live/dead images confirmed the good cytocompatibility of the formulation, with the highly porous structure allowing for a homogeneous diffusion of nutrients, which enhanced cell survival. Results from metabolic activity suggest an improvement from day 1 to 7 in CHAF_1 hydrogel. Interestingly, a boost in cell viability leading to cell proliferation could be attributed to HA properties [64].

These findings indicate that this hydrogel formulation has the potential to be used in a wide range of TE applications, including 3D models and wound healing systems. Due to the wounds’ sensitivity to bacterial infections and the short supply of autografts and allografts commonly used to treat them, full-thickness skin defect healing represents a significant challenge [65,66]. To address this issue, due to their bioactive properties, collagen, HA, and fibrin are often used in skin TE. Collagen hydrogels provide hemostasis, moisturizing, and high biocompatibility, all of which are beneficial to cell proliferation and migration to quicken the healing of wounds [67,68]. Additionally, HA-based materials have often been used to deliver agents that can promote wound healing (such as VEGF and fibroblast growth factor) and prevent bacterial infections (such as antibiotics) [69,70,71]. Finally, fibrin hydrogels are widely used to enhance re-epithelisation and speed up the cutaneous wound regenerative process [72]. These fibrin hydrogels maintain cell viability, promote angiogenic activity, and are an optimal vector for growth factor delivery [73,74]. Given that CHAF hydrogel combines these three biomaterials, it can be hypothesized that its suitability in wound healing applications, but further evaluation is required.

Additionally, in this work, CHAF hydrogel has been evaluated in terms of potential applications in 3D tissue culture (for example, to prevent spheroid disruption and support their growth) and potential skin cancer models. For this, melanoma spheroids were obtained using the 96 round-bottom well plate method, which allowed us to obtain the aggregates in an easy and fast way with great morphological features. To be able to complete that, methylcellulose was added to increase the viscosity of the medium, as it is demonstrated to enhance spheroid formation [75]. After 3 days, cell aggregates were clearly visible with the naked eye, and after 4 days from the initial spot, they were ready to be included in the tissue model. As shown in Figure 9a, increasingly higher cell densities correspond to larger spheroid sizes. However, the spheroid area did not grow proportionally with cell density, and it was observed that higher cell amounts produced much denser spheroids. Notably, despite their round morphology, spheroids made of 5000 and 10,000 cells were quite polydispersed, and cellular aggregation was scarce. With higher cell densities (25.000 and 50.000 cells/spheroid), cells aggregated easily with perfectly rounded shapes that remained stable over time. Spheroids with diameters greater than 500 µm develop typical features of tumor tissues due to the lack of oxygen and nutrients supply to the core; for the purposes of this work, bigger spheroids were considered optimal to mimic tumor metastasis [76]. Additionally, it can be observed from Figure 9a that spheroids reach a higher level of compactness from day 3 to day 7, and this is particularly evident in 25,000 and 50,000-cell spheroids due to stronger cell–cell interactions. In fact, compactness refers to the degree of remodeling that occurs when cells secrete ECM proteins after coming into contact [77]. Moreover, some studies have linked the increase in the hypoxic center inside the spheroids with the compactness level of the spheroids [78]. It was also observed that with higher tumor cell content, the margins of the produced spheroids are rougher, with cells sprouting or detaching from the aggregate. These results may suggest a higher invasive potential, also enhanced by the fact that the employed cells originate from the metastatic growth phase. However, considering the limited dimensions of the model, the best compromise between all requirements resulted in using an intermediate cell density of 25,000 cells/spheroid whose diameter was approximately 800 µm. Notably, the passage of the cells had an influence on spheroid formation, which did not occur after passage 43. It was hypothesized that a correlation between late passages and cell migratory behavior might have affected cell spheroid formation. This hypothesis is supported by previous studies that linked high cell passages with spheroids formation and changes in cell migratory patterns [79]. At day 4 of spheroids maturation, they were embedded into CHAF_1 hydrogel. As observed in Figure 9a,c, spheroids embedded in CHAF_1 maintained their round shape, which is an important feature considering the future drug screening uses of this melanoma model. The preservation of spheroid morphology is a key requirement, given that the outcomes of treatment are often predicted through a size-based analysis. [80]. The spheroid viability and morphology were maintained over time, as observed in the live/dead stained images (Figure 9c). Still, the formation of a hollow apoptotic core was found (cells stained red) at the center of each aggregate. Cell viability within the spheroids was further assessed through CellTiter-Glo^®^ 3D assay, which determines the number of viable cells by quantifying the ATP present. As observed in Figure 7d, the increase in cell viability from day 1 to 3 of spheroids embedded in CHAF_1 hydrogel suggests CHAF_1 suitable composition to support cellular functions. For example, the presence of HA chemical clues to melanoma cells (e.g., cell surface receptors, such as CD44, which is the principal HA receptor on melanoma cells) and increased concentration can modulate tumor progression and poor survival rates in melanoma [81]. Several studies report that HA has been associated with melanoma cell proliferation, adhesion, invasion, and metastatic potential [82]. 

Considering other applications of the hydrogel, its properties would need to be further optimized by changing the crosslinker or the concentrations of components, and for in vivo applications, additional testing would be required to validate the obtained formulation. This study aimed at providing a proof-of-concept for culturing melanoma spheroids; nevertheless, for an in vitro model, it will need to be refined, further established, and validated. Also, the validation of this model as a drug screening platform should be performed by the administration of currently used clinical drugs, and preclinical studies may be conducted on spheroids generated from melanoma cells freshly isolated from patients, paving the way to more personalized medicine.

As future progress, the epidermal compartment can be established by seeding keratinocytes at the air–liquid interface on the top of the dermal compartment. As previously mentioned, the formulation may be used as a bio-ink, and this would allow a precise deposition of different cell types to closely resemble human skin. Furthermore, the tumor microenvironment in vivo is even more complex than the one proposed here, harboring several tumor-associated cell types, such as immune cells, whose inclusion might be useful for immunotherapy testing. 

## 5. Conclusions

A new ECM bioinspired tri-component hydrogel has been formulated and synthesized by exploiting collagen type I, hyaluronic acid, and fibrinogen to mimic the skin composition. The formulated hydrogels demonstrated enhanced viscoelastic properties with increased concentrations of 4S-StarPEG as a crosslinker, showing crosslinking degrees over 60%. The CHAF_1 evidenced suitable viscoelastic properties that closely resembled native skin, enhanced porous structure, and cytocompatibility when tested with human fibroblasts. In addition, the formulated hydrogel was demonstrated to facilitate the growth of melanoma spheroids, maintaining their 3D shape and integrity while favoring their growth when cultured over 7 days. Together, these results evidenced how formulated ECM biomimetic hydrogel offers the scientific community a bio-functional matrix for soft tissue engineering applications. From this work, it is anticipated that the ability to tune the gelation time and viscoelastic properties of formulated hydrogels will allow researchers to exploit these into a wide range of applications, spanning from (but not limited to) in vitro tissue models to study cell interactions, understand diseases, test drugs, 3D tissue culture of spheroids and organoids, up to bioprinting.

## Figures and Tables

**Table 1:** The nomenclature used for the different hydrogel formulations and the collagen to the 4S-StarPEG molar ratio used.

Sample	Crosslinker
CHAF_1	PEG (C/PEG 1:1)
CHAF_10	PEG (C/PEG 1:10)
CHAF_50	PEG (C/PEG 1:50)

**Table 2:** Storage (G′) and loss (G″) moduli values in kPa for the three different CHAF hydrogel formulations.

Sample	G′ (kPa)	G″ (kPa)
CHAF_1	37.6	9.1
CHAF_10	78.1	16.0
CHAF_50	64.5	10.7

**Table 3:** IR characteristic peaks of all the materials and crosslinker of CHAF_1 and CHAF_1.

Material Spectrum	Wavenumber (cm^−1^)	Chemical Bond
Collagen	1657	α-helix secondary structure [30] C=O stretching [31]
	1555	N–H bending vibration [32] C–N stretching vibration [32]
	1403	C=O stretching of COO– [33]
	1240	C–N stretching [33]
	1161, 1066	CO stretching
	860	CH bending
Hyaluronic Acid	1738, 1617	Amide I [34]
	1560	Amide II [34]
	1325	Amide III [35]
	2922	C–H stretching
	1082, 1043	C–O, C–C, and C–OH groups [35]
	944	Carbohydrates [36]
Fibrin	1700–1600	Amide I [37]
	1643, 1534	Amide II [38,39]
	1314	Amide III
	1077	CO stretching [33]
4S-StarPEG	2885	–CH_2_- bonds [40]
	1100	C–O–C bonds [40]
	1740	Succinimidyl ester C=O stretch
	1467	C–H stretching of methylene group of alkyl chain
	1342, 1241	Ester bonds
	1147	C–O–C stretching
	1060	C–O stretching
	961, 843	C–H bending [41]
	1700–1600	Amide I [37]
	1557	Amide II
	1640	NH–C=O (amide bond formation)
	3320	–OH groups
	1366	C=O stretching
	1230	Amide bond

**Table 4:** Comparison of percentage of water content (CHAF_1 and human dermis [42]).

Material	Water Content (%)
Human dermis	70–75
CHAF_1	71.8 ± 12.7

**Table 5:** Average pore size through the degradation test of CHAF_1. Data indicate mean ± SD.

Pore Size		
Day 0	Day 2	Day 7
120 ± 97 μm	131 ± 54 μm	169 ± 82 μm

## Data Availability

Data supporting this publication are openly available at https://data.ncl.ac.uk/ (accessed on 10 August 2023).
